# Toxic Acetaminophen Exposure Induces Distal Lung ER Stress, Proinflammatory Signaling, and Emphysematous Changes in the Adult Murine Lung

**DOI:** 10.1155/2019/7595126

**Published:** 2019-11-28

**Authors:** Jeryl Sandoval, David J. Orlicky, Ayed Allawzi, Brittany Butler, Cynthia Ju, Caroline T. Phan, Roy Toston, Robyn De Dios, Leanna Nguyen, Sarah McKenna, Eva Nozik-Grayck, Clyde J. Wright

**Affiliations:** ^1^Section of Neonatology, Department of Pediatrics, University of Colorado School of Medicine, Aurora, CO, USA; ^2^Department of Pathology, University of Colorado School of Medicine, Aurora, CO, USA; ^3^Developmental Lung Biology and Cardiovascular Pulmonary Research Laboratories, Departments of Pediatrics and Medicine, University of Colorado, Anschutz Medical Campus, Aurora, CO, USA; ^4^Department of Anesthesiology, The University of Texas Health Science Center at Houston, McGovern Medical School, Houston, TX, USA

## Abstract

Clinical studies have demonstrated a strong association between both acute toxic exposure and the repetitive, chronic exposure to acetaminophen (APAP) with pulmonary dysfunction. However, the mechanisms underlying this association are unknown. Preclinical reports have demonstrated that significant bronchiolar injury occurs with toxic APAP exposure, but very little information exists on how the distal lung is affected. However, cells in the alveolar space, including the pulmonary epithelium and resident macrophages, express the APAP-metabolizing enzyme CYP2E1 and are a potential source of toxic metabolites and subsequent distal lung injury. Thus, we hypothesized that distal lung injury would occur in a murine model of toxic APAP exposure. Following exposure of APAP (280 mg/kg, IP), adult male mice were found to have significant proximal lung histopathology as well as distal lung inflammation and emphysematous changes. Toxic APAP exposure was associated with increased CYP2E1 expression in the distal lung and accumulation of APAP-protein adducts. This injury was associated with distal lung activation of oxidant stress, endoplasmic reticulum stress, and inflammatory stress response pathways. Our findings confirm that following toxic APAP exposure, distal lung CYP2E1 expression is associated with APAP metabolism, tissue injury, and oxidant, inflammatory, and endoplasmic reticulum signaling. This previously unrecognized injury may help improve our understanding of the relationship between APAP and pulmonary-related morbidity.

## 1. Introduction

Acetaminophen (*N*-acetyl-p-aminophenol, APAP) is the most commonly used analgesic and antipyretic around the world [1, 2]. APAP is perceived to be safe and effective, leading to human exposures that begin in utero with maternal use and continue throughout lifetime. However, this perception is challenged by the fact that acute overdose of APAP is highly toxic and is the leading cause of acute liver failure in the USA and Europe [1, 2]. Additionally, it is increasingly recognized that chronic APAP use is associated with various morbidities affecting multiple-organ systems [3, 4].

One consistent observation has been the association between APAP exposure and pulmonary dysfunction. Both prenatal and early-life APAP exposures have been linked to an increased risk of developing asthma [3, 5–7]. Furthermore, acute overdose has been associated with pulmonary injury [8, 9]. Preclinical studies have also linked APAP exposure to lung injury. Rats and mice exposed to APAP develop bronchiolar injury that occurs within 4 hours of initial exposure [10–14]. When looked for, pulmonary injury induced by toxic APAP exposure occurs as consistently and with similar severity as hepatic injury [13, 15]. Despite this consistent and reliable observation, the mechanisms underlying APAP-induced pulmonary injury are unknown [4].

In contrast, the mechanisms underlying APAP-induced liver injury have been well studied. In response to toxic APAP exposure, the hepatic cytochrome P450 2E1 (CYP2E1) enzyme converts APAP to its toxic metabolite N-acetyl-para-benzo-quinone imine (NAPQI). Accumulation of this toxin leads to multiple deleterious effects, including glutathione (GSH) depletion, oxidative damage, APAP-protein adducts, mitochondrial dysfunction, and hepatocyte death [2, 16, 17]. A growing body of literature supports the hypothesis that these same mechanisms are also active in the lung, making it susceptible to the toxic effects of APAP. Direct intratracheal administration of the toxic APAP metabolite NAPQI injures the trachea and bronchial epithelium [18]. Importantly, like the liver, the lung expresses the APAP-metabolizing enzyme CYP2E1. Previous studies have demonstrated that the bronchial and bronchiolar epithelia [19–22], as well as the club cell within the bronchioles [11], express CYP2E1. Accordingly, following APAP exposure, APAP-protein adducts can be detected in the areas of the lung that express CYP2E1 [11, 15, 18, 23, 24]. These data support the hypothesis that pulmonary metabolism of APAP results in localized GSH depletion and accumulation of toxic metabolites resulting in injury to the large conducting airways.

Importantly, expression of the APAP-metabolizing enzyme CYP2E1 is not limited to the proximal lung and larger conducting airways. Specifically, CYP2E1 is also expressed in the peripheral lung [25], including the alveolar epithelium and alveolar macrophages [26, 27]. Furthermore, APAP exposure is toxic to isolated type II pulmonary epithelial cells and alveolar macrophages and associated with GSH depletion [9, 28]. However, the mechanism by which this injury occurs and whether APAP exposure damages the distal lung *in vivo* is unknown. Understanding whether the distal lung is susceptible to the toxic effects of APAP would improve our understanding of the mechanisms underlying APAP exposure and long-term pulmonary dysfunction.

Therefore, we hypothesized that distal lung injury would occur in a murine model of toxic APAP exposure. In this study, we exposed adult male mice to APAP (280 mg/kg, IP) and performed robust and blinded histopathologic assessments of pulmonary injury. We found that in addition to significant proximal lung injury with epithelial cell death, toxic APAP exposure induced distal lung inflammation and emphysematous changes. Concurrently, we observed activation of proinflammatory and endoplasmic reticulum (ER) stress response signaling. Immunofluorescent staining confirmed CYP2E1 expression in the distal lung, and the presence of CYP2E1 in the distal lung was confirmed via Western blot of isolated microsomes. Importantly, following toxic APAP exposure, APAP adducts were present in the areas of distal lung injury. This injury was associated with GSH depletion and activation of proinflammatory NF*κ*B signaling and target gene expression. Our findings confirm that following toxic APAP exposure, distal lung CYP2E1 expression is associated with APAP metabolism and tissue injury as well as inflammatory and ER signaling. This previously unrecognized injury may help explain the association between APAP exposure and pulmonary morbidity and suggest the importance of further studies. These results also urge cautious use of APAP in settings where the effect on the lung is unknown and understudied.

## 2. Methods

### 2.1. Murine Model of Toxic Acetaminophen (APAP) Exposure

Adult (6–8-week old) male ICR mice were exposed to APAP (280 mg/kg, intraperitoneal (IP); dissolved in phosphate-buffered saline). Mice were fasted overnight (~16 hours) prior to the day of exposure. Following exposure, the mice were sacrificed, blood was collected by cardiac puncture after which normal saline was perfused through the right ventricle, and tissue samples were collected and processed as described below. All procedures were approved by the IACUC at the University of Colorado (Aurora, CO), and care and handling of the animals was in accord with the National Institutes of Health guidelines for ethical animal treatment.

### 2.2. Histologic Evaluation of APAP-Induced Hepatic Injury

Liver tissue was fixed with 4% paraformaldehyde, paraffin embedded, and sections were cut (5 *μ*m) and stained with hematoxylin and eosin at the University of Colorado Denver Morphology and Phenotyping Core. Histopathological scoring of the liver tissue was performed by a trained histologist blinded to the treatments or grouping of animals. Briefly, the acetaminophen-induced liver injury system relied on 3 semiquantitative and 1 quantitative criteria. These criteria included (1) the extent and locale of necrosis, (2) the extent of inflammatory cell infiltration, (3) the extent of centrilobular sinusoidal dilatation, and (4) measurement of the serum alanine transaminase (ALT) all as indicated in Martin-Murphy et al. [29].

### 2.3. Serum Alanine Aminotransferase (ALT) and HMGB1 Measurements

Serum ALT was quantitatively determined using the ALT (SGPT) reagent and the colorimetric, endpoint method according to the manufacturer's instructions (Teco Diagnostics). Serum HMGB1 was measured using HMGB1 ELISA according to the manufacturer's protocol (MyBioSource).

### 2.4. Histologic Evaluation of APAP-Induced Pulmonary Injury

Following perfusion of the lungs through the right ventricle as described above, the trachea was cannulated with a 24 G angiocatheter. The lungs were inflation-fixed at 20 cm H_2_O pressure for 20 minutes with 4% paraformaldehyde and stored. The lungs were paraffin-embedded, and sections were cut (5 *μ*m) and stained with hematoxylin and eosin at the University of Colorado Denver Morphology and Phenotyping Core. Measurements of mean linear intercept (MLI), a measurement of the mean distance in the air spaces, and airspace area (ASA) and surface area (SA) were performed using the computer-assisted image-analysis program MetaMorph Basic (Molecular Devices, Sunnyvale, CA) with custom-designed macros (subroutines) on images captured on an Olympus IX83 microscope (10x, 20x, and 40x objective lenses). Ten randomly selected nonoverlapping sections per mouse at 40x magnification were assessed.

Histopathological scoring of the lungs was performed by a trained histologist blinded to the treatments or grouping of animals. Briefly, 4 semiquantitative criteria were used for this scoring: (1) the integrity of the respiratory and terminal bronchiole epithelium (0-3, normal to severe, as well as the presence or absence of the apoptotic epithelium in the airway lumen); (2) the relative quantity of bronchus-associated lymphoid tissue (BALT; 0-2, none to lots); (3) the quantity of macrophages found in the peripheral airway (0-3, none to lots with clumps); and (4) the presence of peripheral lung emphysema with alveolar wall clubbing (0-2, none to lots). The relative quantity of neutrophils in the tissue was also noted. Scores were tallied, grouped, and finally unblinded by the individual with knowledge of the groupings. Histological images were captured on an Olympus BX51 microscope equipped with a 17 MP Olympus DP73 high-definition, color, digital camera using the Olympus cellSens software (Olympus, Waltham, MA, USA). All composite images were cropped and assembled using Adobe Photoshop.

### 2.5. Bronchoalveolar Lavage Fluid Analysis

After exposure to APAP, adult mice were sacrificed and bronchoalveolar lavage fluid (BALF) was obtained and analyzed [30]. Total protein content was determined by the Bradford method with bovine serum albumin as a standard. BALF total cell count and cell differentials were assessed for characteristics of pulmonary injury.

### 2.6. Isolation of mRNA, cDNA Synthesis, and Analysis of Relative mRNA Levels by RT-qPCR

At the time of tissue collection, both the right and left lungs were dissected into “proximal” and “distal” sections. The “proximal” section was demarcated by where the conducting airway was no longer visualized without assistance. The “distal” lung was the remainder of the available section. Frozen tissue was placed in RLT buffer (Qiagen), and tissue was homogenized using the Bullet Blender (Next Advance). Pulmonary mRNA was collected from homogenized tissue using the RNeasy Mini Kit (Qiagen) according to the manufacturer's instructions. Initially, tissue RNA was assessed for purity and concentration using the NanoDrop (Thermo Fisher Scientific), and cDNA was synthesized using the Verso cDNA Synthesis Kit (Thermo Fisher Scientific). Relative mRNA levels were evaluated by quantitative real-time PCR using exon spanning primers (Table 1), TaqMan gene expression, and the StepOnePlus Real-Time PCR System (Applied Biosystems). Relative quantitation was determined via normalization to the endogenous control 18S using the cycle threshold (ΔΔCt) method.

### 2.7. Preparation of Whole Lung Lysate and Cytosolic/Nuclear Extracts and Western Blot

Frozen distal pulmonary tissue was homogenized using the Bullet Blender (Next Advance). Pulmonary whole cell lysates were collected in T-PER (Thermo Fisher Scientific), and cytosolic and nuclear extracts were collected in NE-PER (Thermo Fisher Scientific). Lysates and cytosolic and nuclear extracts were electrophoresed on a 4-12% polyacrylamide gel (Invitrogen), and proteins were transferred to an Immobilon Membrane (Millipore) and blotted with antibodies (Table 2). Blots were imaged using the LI-COR Odyssey Imaging System, and densitometric analysis was performed using Image Studio (LI-COR). In the figures, cropped images grouped together are from the same gel. No images have been spliced together and no images from separate blots have been grouped together.

### 2.8. Isolation of Microsomes from Pulmonary Tissue

Microsomes were isolated from distal pulmonary tissue using a microsome isolation kit (Abcam, 206995) according to the manufacturer's instructions.

### 2.9. Immunohistochemical Staining of CYP2E1 and APAP Adducts

For immunofluorescence, tissues were deparaffinized by heating and rehydrated with xylene and ethanol. Antigen retrieval was performed with citrate antigen retrieval buffer, pH 6.0, and tissues were permeabilized with 0.5% triton, quenched with glycine, and blocked with 5% donkey serum. Sections were then incubated with anti-acetaminophen (Bio-Rad, 0016-0104) and anti-CYP2E1 (Abcam, ab28146) primary antibodies overnight followed by incubation with secondary antibodies (Alexa Fluor Anti-Rabbit Donkey 594 and Alexa Fluor Anti-Sheep 647) for 1 hour. Finally, nuclear counterstaining was performed by incubating slides with DAPI (Sigma-Aldrich, D8417). Staining was visualized using the Olympus IX83 microscope and Olympus DP80 camera using Olympus cellSens software.

### 2.10. Pulmonary and Hepatic Glutathione Fluorescent Detection

Pulmonary and hepatic whole cell lysates were collected in T-PER (Thermo Fisher Scientific). Lysate extracts were serially diluted, and glutathione (GSH) and oxidized glutathione (GSSG) contents were quantified using the Glutathione Fluorescent Detection Kit (Thermo Fisher Scientific).

### 2.11. Flow Sorting

Lungs were digested as previously described [31]. In brief, lungs were first inflated with CD45-APC (Clone # 30-F11) at a concentration of 1/500 to label alveolar leukocytes for 3 minutes. Lungs were then flushed and digested with Liberase™ (0.4 mg/ml) and DNAse I (100 U/ml) by mincing in a gentleMACS C tube using program m_lung_01_02 for a full cycle, incubated for 20 minutes at 37°C, then agitated again using program m_lung_02 for 5 seconds of the cycle. Cell suspension was then washed through a 70 and 40 *μ*m cell strainer and red blood cell (RBC) lysis was performed using 1x RBC Lysis Buffer (eBioscience). Cell surface staining was then performed using the following antibodies: Ly6G-BV421 (Clone # 1A8), CD3-BV421 (Clone # 17A2), Ly6C-BV421 (Clone # AL-21), CD31-BV421 (Clone # MEC13.3), CD326-BV510 (Clone # G8.8), CD64-PE (Clone # X54-5/7.1), CD11b-PE/Cy7 (Clone # M1/70), and SigF-APC/R700 (Clone # E50-2440). All antibodies were used at a concentration of 1/200 and stained on ice for 15 minutes. Cells were then washed using 1 ml of cell staining buffer and centrifuged at 300 rcf for 5 minutes. Cell pellets were then resuspended with 500 *μ*l of 1x Cytofix buffer (Biolegend) and incubated in the dark at room temperature for 30 minutes. Cells were then washed with 1 ml of Cytoperm wash buffer and centrifuged at 300 rcf for 5 minutes then resuspended in 100 *μ*l of Cytoperm wash buffer and stained with CYP2E1-AF488 antibody overnight at 4°C. Cells were washed using 1 ml of cell staining buffer and then analyzed on a ZE5 Cell Analyzer (Propel Labs). Single cells were identified using forward and side scatter followed by forward scatter area and height. Using a dump gate, monocytes, neutrophils, B-cells, and T-cells were excluded and macrophages were identified using CD45 and CD64. Resident and recruited macrophages were differentiated using positive selection for SigF and CD11b, respectively. Epithelial cells were gated on using positive staining for CD326 and negative staining for CD45. CYP2E1 expression was performed by assessing geometric mean fluorescence (MFI) for resident macrophages (Dump^−^, CD45^+^CD64^+^SigF^+^CD11b^−^), recruited macrophages (Dump^−^, CD45^+^CD64^+^CD11b^+^SigF^−^), and epithelial cells (Dump^−^, CD45^−^CD326^+^).

### 2.12. Statistical Analysis

For comparison between treatment groups, the null hypothesis that no difference existed between treatment means was tested by Student's *t*-test for two groups and two-way ANOVA for multiple groups with potentially interacting variables (time, APAP exposure), with statistical significance between and within groups determined by means of the Bonferroni method of multiple comparisons (Prism, GraphPad Software, Inc.). Statistical significance was defined as *p* < 0.05.

## 3. Results

### 3.1. Time Course of APAP-Induced Hepatic Injury in ICR Mice

**Figure 1:**
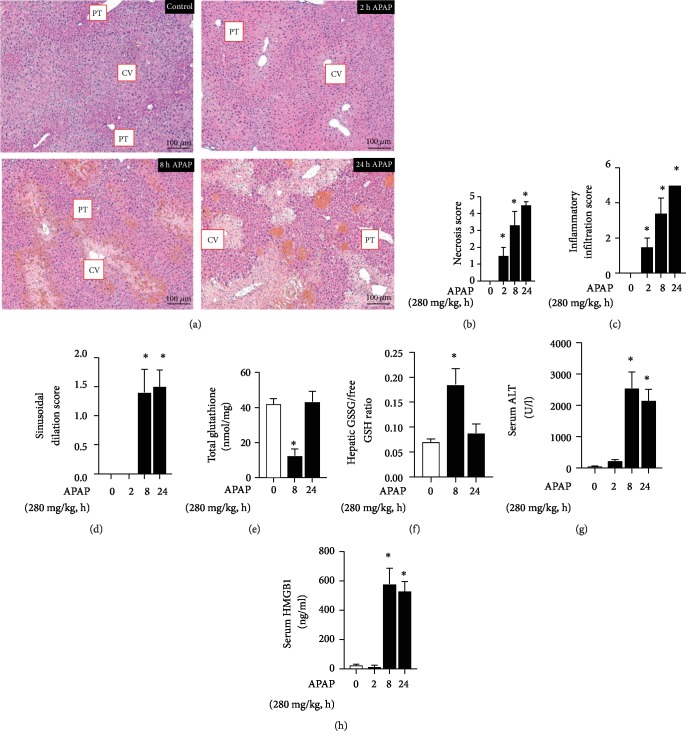
Time course of APAP-induced hepatic injury in ICR mice. (a) Representative H&E-stained hepatic sections from control and APAP-exposed (2, 8, and 24 hours; 280 mg/kg, IP) adult male ICR mice. Examples of portal triad (PT) and central vein (CV) have been added. Internal scale bar: 100 *μ*m. (b–d) Blind histopathologic evaluation of H&E-stained hepatic sections scored for (b) necrosis, (c) inflammatory infiltration, and (d) sinusoidal dilatation. *N* = 6‐8 per time point. Data are expressed as mean ± SEM; ^∗^*p* < 0.05 vs. unexposed control. (e) Total hepatic glutathione, (f) ratio of oxidized (GSSG) vs. reduced free glutathione (GSH), and change in serum (g) ALT and (h) HMGB1 protein following APAP exposure (280 mg/kg, IP). *N* = 6‐8 per time point. Data are expressed as mean ± SEM; ^∗^*p* < 0.05 vs. unexposed control.

First, we sought to confirm the time course of APAP-induced liver injury in adult male ICR mice. Histologic analysis demonstrated necrotic and inflammatory injury as soon as 2 hours after APAP exposure (Figure 1(a)). Blinded histopathologic analysis revealed early and significant increases in objective scoring of necrosis (Figure 1(b)) and inflammation (Figure 1(c)) that were sustained from 2 hours through 24 hours post APAP exposure, while sinusoidal dilatation was significantly increased at 8 and 24 hours of exposure (Figure 1(d)). Concurrent with histologic evidence of injury, hepatic total glutathione decreased (Figure 1(e)) and GSSG/GSH ratio increased (Figure 1(f)). Finally, there was a significant increase in circulating markers of injury, including serum ALT (Figure 1(g)) and serum HMGB1 (Figure 1(h)). These data reliably demonstrate that significant hepatic injury occurs early and is sustained during the first 24 hours following an IP exposure to APAP.

### 3.2. Toxic APAP Exposure Induces Proximal and Distal Lung Injury

**Figure 2:**
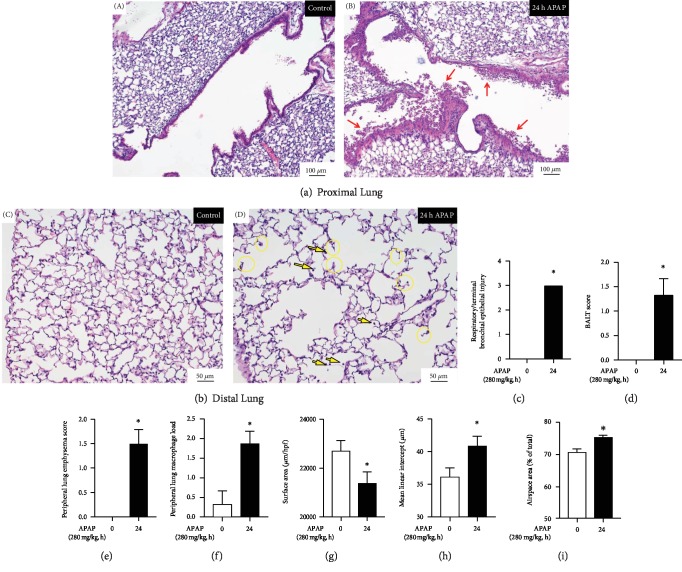
Toxic APAP exposure induces proximal and distal lung injury. Representative H&E-stained (a) proximal control (A) and APAP-exposed (B) (24 hours, 280 mg/kg, IP) adult male ICR mice and (b) distal control (C) and APAP-exposed (D) (24 hours, 280 mg/kg, IP) adult male ICR mice. Red arrows indicate epithelial cells in the airway lumen. Yellow arrows indicate distal lung macrophages. Yellow circles indicate emphysematous-like changes of the breakdown of alveolar walls and the “clubbing” of the broken wall tops. (c) Respiratory/terminal bronchiole epithelial injury, (d) bronchus-associated lymphoid tissue (BALT), (e) peripheral lung emphysema score, (f) peripheral lung macrophage load, (g) surface area, (h) mean linear intercept, and (i) airspace area in control vs. APAP-exposed (280 mg/kg, IP) adult male ICR mouse lung. *N* = 3‐4 per time point. Data are expressed as mean ± SEM; ^∗^*p* < 0.05 vs. unexposed control.

Next, we performed histopathologic evaluation of the lungs of APAP-exposed mice. Consistent with previous reports, APAP exposure induced significant injury to the proximal airway including death and shedding of some of the injured pseudostratified columnar epithelium into the airway lumen (Figure 2(a) B, red arrows). Objective scoring showed a significant increase in respiratory and terminal bronchial epithelial injury (Figure 2(c)) and bronchus-associated lymphoid tissue (BALT, Figure 2(d)) at 24 hours of APAP exposure. In addition to this bronchiolar injury, we observed significant changes in the alveolar lung structure that included the emphysematous-like changes of breakdown of alveolar walls and “clubbing” of the broken alveolar wall tops (Figure 2(b) D, yellow circles). Additionally, the luminally located alveolar macrophage load increased (Figure 2(b) D, yellow arrows). Objectively, this manifested as an increase in the peripheral lung emphysema score (Figure 2(e)) and the peripheral lung airway macrophage load (Figure 2(f)). Objective morphometric analysis revealed that APAP exposure resulted in decreased surface area (Figure 2(g)), increased mean linear intercept length (Figure 2(h)), and increased airspace area (Figure 2(i)), with all markers being consistent with a destructive type of alveolar injury.

**Figure 3:**
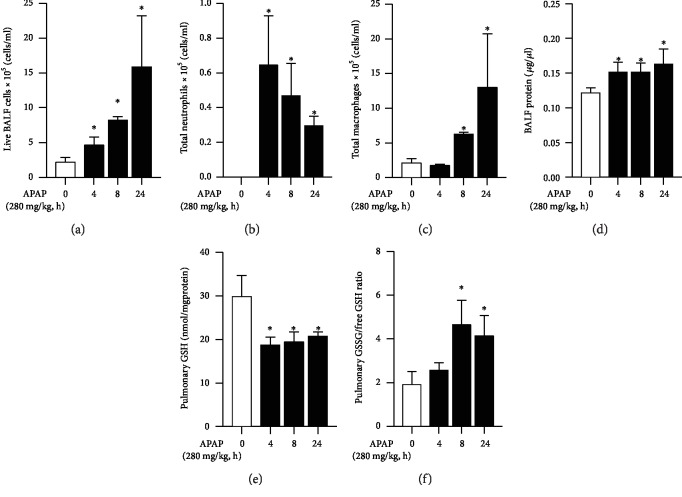
Toxic APAP exposure induces pulmonary inflammation and oxidant stress. Bronchial-alveolar fluid lavage (BALF) analysis of (a) live BALF cells, (b) total neutrophils, (c) total macrophages, and (d) BALF protein. (e) Total pulmonary glutathione and (f) ratio of oxidized (GSSG) vs. reduced free glutathione (GSH) following APAP exposure (4, 8, and 24 hours; 280 mg/kg, IP) in adult male ICR mice. *N* = 6‐8 per time point. Data are expressed as mean ± SEM; ^∗^*p* < 0.05 vs. unexposed control.

Having observed this injury, we next evaluated the bronchial-alveolar lavage fluid for evidence of injury. Analysis of BALF fluid obtained from mice exposed to APAP revealed additional markers of injury. The total cellular content of BALF significantly increased in a time-dependent manner following APAP exposure (Figure 3(a)), with an initial increase in neutrophils (Figure 3(b)) followed by macrophages (Figure 3(c)). The total protein content of BALF fluid significantly increased at 4 hours of exposure and remained at this elevated level throughout 24 hours of exposure (Figure 3(d)). Concurrent with histologic and BALF evidence of injury, the lungs of APAP-exposed mice demonstrated evidence of oxidant stress and glutathione depletion consistent with toxic APAP exposure. Specifically, pulmonary total glutathione remained significantly decreased (Figure 3(e)) and GSSG/GSH ratio remained significantly increased (Figure 3(f)) through the duration of exposure. Together, these data demonstrate that alveolar injury occurs along with the injury previously described in the larger conducting airways.

### 3.3. The Distal Lung Expresses CYP2E1

**Figure 4:**
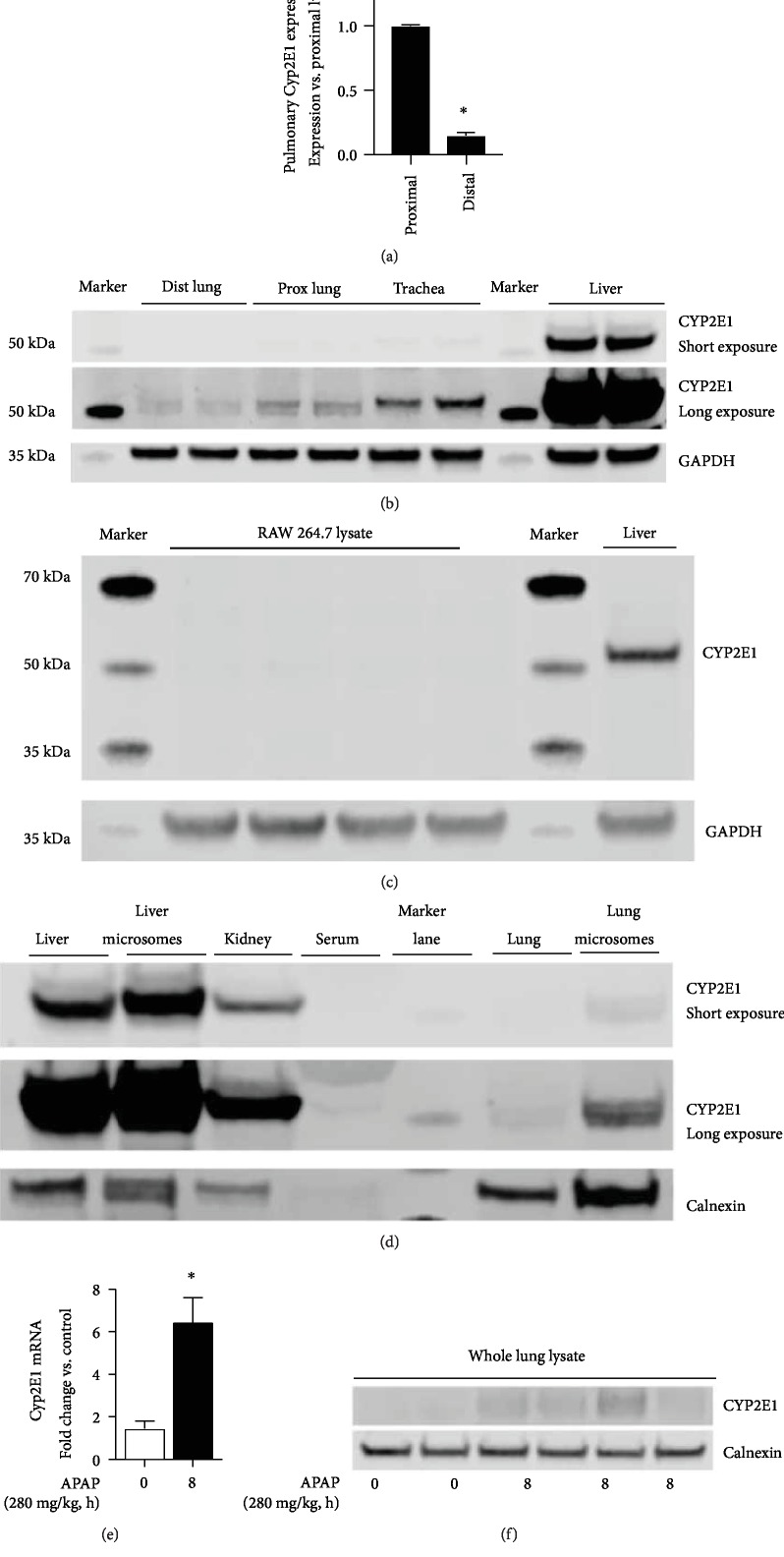
The distal lung expresses CYP2E1. (a) Fold difference in CYP2E1 mRNA expression in the distal compared to the proximal lung. Data are expressed as mean ± SEM; ^∗^*p* < 0.05 vs. proximal lung. *N* = 6‐8 mice. (b) Representative Western blot of CYP2E1 in the distal and proximal lung, as well as the trachea and liver. A “short exposure” is provided to demonstrate CYP2E1 expression in the liver without overexposing the blot. The “long exposure” is provided to allow comparisons between the distal lung, proximal lung, and trachea. GAPDH as loading control. (c) Representative Western blot of CYP2E1 in the RAW 264.7 whole cell lysate and liver homogenate. GAPDH as loading control. (d) Representative Western blot of CYP2E1 in the liver, liver microsomes, kidney, serum, lung, and lung microsomes. For each sample, 40 *μ*g of protein was evaluated. Calnexin as loading control, to demonstrate enrichment of the microsomal fraction. (e) Fold change in CYP2E1 mRNA expression in the distal lung following APAP exposure (8 hours; 280 mg/kg, IP). Data are expressed as mean ± SEM; ^∗^*p* < 0.05 vs. control. *N* = 4‐6 per time point. (e) Representative Western blot of whole lung lysate following APAP exposure (8 hours; 280 mg/kg, IP). Calnexin shown as loading control.

Next, we sought to answer whether the APAP-metabolizing enzyme CYP2E1 was expressed in the distal lung. While the expression of CYP2E1 in the alveolar space of humans has been described, expression in the murine lung is less well characterized [25–27]. At baseline, and prior to any APAP exposure, we found that the distal lung expressed *Cyp2E1* mRNA, albeit at a level significantly lower than the proximal lung (Figure 4(a)). Protein expression in whole lung homogenate isolated from proximal and distal lung mirrored mRNA expression, with CYP2E1 being more highly expressed in the proximal lung (Figure 4(b)). Comparatively, there is a higher expression in the trachea, and expression in the liver far exceeds that of the distal and proximal lung (Figure 4(b)). The antibody for CYP2E1 used for these experiments performed as expected with negative controls (RAW 264.7) and positive controls (liver homogenate) (Figure 4(c)). Furthermore, the antibody detects higher expression of CYP2E1 in liver microsomes compared to liver homogenate, higher expression in the liver compared to that in the lungs, higher expression in lung microsomes compared to that in lung homogenate, expression in the kidney, and no cross reactivity with serum proteins (Figure 4(d)). Importantly, following APAP exposure, significant increases in distal lung *Cyp2E1* mRNA expression were observed (Figure 4(e)). Additionally, whole lung CYP2E1 content increased following APAP exposure (Figure 4(f)). These data confirm that at the location of injury—specifically the alveolar space—the APAP-metabolizing enzyme CYP2E1 is expressed and that this expression increases in the lung following toxic APAP exposure.

### 3.4. Toxic APAP Metabolites and CYP2E1 Expression Localizes to the Distal Lung

**Figure 5:**
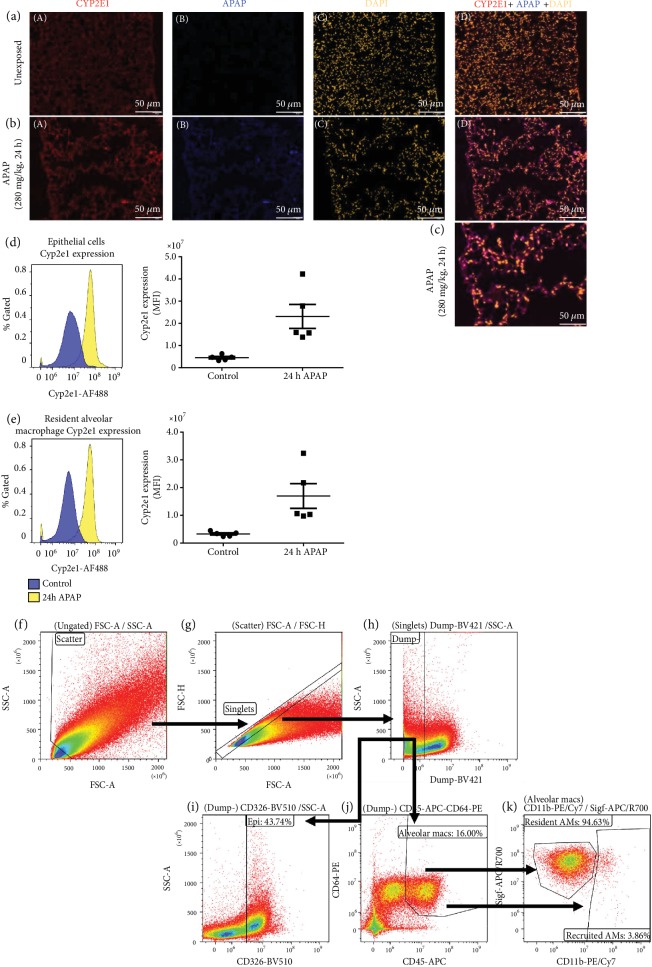
Toxic APAP metabolites accumulate in the distal lung. Representative immunofluorescence staining of the distal lung from (a) unexposed and (b and c) APAP-exposed (24 hours; 280 mg/kg, IP) adult male ICR mice. CYP2E1 was stained in red (a), APAP was stained in blue (b), and DAPI was stained in yellow (c). An overlay of all three stains (a, b, and c) is provided (d). Internal scale bar: 50 *μ*m. Flow cytometric analysis demonstrates an induction of CYP2E1 expression in APAP-exposed mice when compared to unexposed controls in (d) epithelial cells and (e) resident alveolar macrophages. (f–k) Flow gating strategy. (f) Scatter cells are gated into (g) singlets, after which (h) neutrophils, B-cells, T-cells, and monocytes are excluded and the Dump^–^ gate is used to select for (i) epithelial (Dump^−^, CD326^+^) or (j) alveolar macrophages (Dump^−^, CD45^+^CD64^+^). (k) Resident (Dump^−^, CD45^+^CD64^+^Siglec-F^Hi^CD11b^Lo^) and recruited (Dump^–^, CD45^+^CD64^+^CD11b^Hi^Siglec-F^Lo^) alveolar macrophages are then segregated based on Siglec-F and CD11b.

Having determined that the distal lung expresses CYP2E1, we assessed the presence of APAP-protein adducts using fluorescent immunostaining. Consistent with our results from qPCR and Western blot, the distal lung demonstrated CYP2E1 staining that was present prior to (Figure 5(a) A) and after (Figure 5(b) A) APAP exposure. Of note, at 24 h post APAP exposure, alveolar staining was diffused and punctuated with positive staining in larger cells in the lumen of the alveoli and the alveoli show the emphysematous changes described above. Importantly, APAP-protein adduct staining that was absent in the unexposed lung (Figure 5(a) B) appeared in the distal lung following APAP exposure (Figures 5(b) B and 5 (c)). To further determine the cellular expression of CYP2E1, we used intracellular flow cytometry to assess CYP2E1 expression in epithelial cells and macrophages in the lungs of control and APAP-exposed mice. The flow gating strategy is provided (Figures 5(f)–5(k)). In line with our fluorescent immunostaining, CYP2E1 fluorescence is increased in both epithelial (Dump^−^, CD45^−^CD326^+^) (Figure 5(d)) and resident alveolar macrophages (Dump^−^, CD45^+^CD64^+^SigF^+^CD11b^−^) (Figure 5(e)). Intriguingly, there was no presence of a distinct recruited alveolar macrophage population (CD45^+^CD64^+^CD11b^+^SigF^−^) in the lungs of either control or APAP-exposed mice 24 hours post exposure. While histopathologic evaluation of the lung demonstrated an increase in peripheral lung macrophage load at 24 hours of APAP exposure (Figure 2(f)), this increase was not detectable by flow cytometry as an increase in recruited alveolar macrophages. A review of the histology demonstrates a visible increase in the number of peripheral lung macrophages; however, the number of recruited cells appears less than would be present with other types of inflammatory injury (e.g., pneumonia). We speculate this visible increase was not large enough to provide an adequate number of cells to make any conclusions using flow cytometry. These results demonstrate that the APAP-metabolizing enzyme CYP2E1 is expressed and is active in the distal lung and that toxic APAP metabolites accumulate in the distal lung where injury is observed.

### 3.5. Toxic APAP Exposure Induces Proinflammatory NF*κ*B Signaling in the Distal Lung

Toxic APAP exposure is associated with multiple cellular insults that can lead to downstream proinflammatory signaling. These include, but are not limited to ROS production, inflammasome activation and IL1*β* secretion, HMGB1 release, and TLR4/TLR9 signaling [16, 32, 33]. Of note, the transcription factor NF*κ*B is activated downstream of these pathways and is known to control the expression of multiple genes previously identified to be involved in APAP-induced hepatic injury. Thus, we interrogated APAP-induced pulmonary NF*κ*B activation and downstream gene expression.

**Figure 6:**
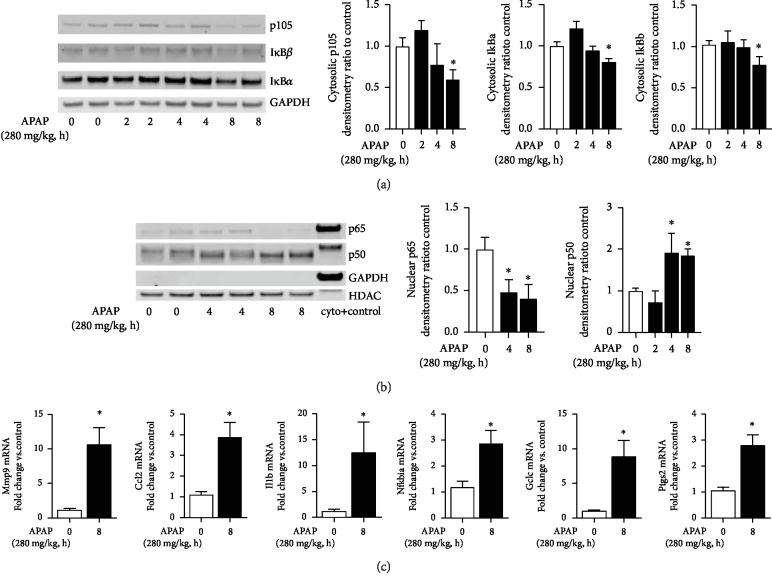
Toxic APAP exposure induces proinflammatory NF*κ*B signaling in the distal lung. (a) Representative Western blot and densitometric analysis of NF*κΒ* inhibitory proteins p105, I*κ*B*α*, and I*κ*B*β* in distal lung cytosolic extracts after APAP exposure (2, 4, and 8 hours; 280 mg/kg, IP). GAPDH is shown as loading control. Data is expressed as mean ± SEM (*N* = 4‐6 per time point). ^∗^*p* < 0.05 vs. unexposed control. (b) Representative Western blot and densitometric analysis of NF*κ*Β subunits p65 and p50 in distal lung nuclear extracts after APAP exposure (2, 4, and 8 hours; 280 mg/kg, IP). HDAC is shown as a loading control. GAPDH is shown to confirm the purity of the nuclear sample. Data is expressed as mean ± SEM (*N* = 4‐6 per time point). ^∗^*p* < 0.05 vs. unexposed control. (c) Fold change in NF*κ*Β target gene mRNA expression: *Mmp9*, *Ccl2*, *Il1b*, *Nfkbia*, *Gclc*, and *Ptgs2* following APAP exposure (8 hours; 280 mg/kg, IP). Data expressed as mean ± SEM (*N* = 4‐6 per time point). ^∗^*p* < 0.05 5 vs. unexposed control.

First, we assessed pulmonary cytosolic extracts from APAP-exposed adult mice for evidence of degradation of the NF*κ*B inhibitory proteins p105, I*κ*B*α*, and I*κ*B*β*. In APAP-exposed adult mice, p105, I*κ*B*α*, and I*κ*B*β* had all degraded by 8 hours of exposure (Figure 6(a)). We next sought to confirm that cytosolic NF*κ*B inhibitory protein degradation was associated with pulmonary nuclear translocation of the NF*κ*B subunits p65 and p50. In APAP-exposed mice, there was robust nuclear accumulation of p50 while levels of p65 decreased and were nearly absent by 8 hours (Figure 6(b)). Interestingly, this pattern of NF*κ*B subunit nuclear translocation mirrors what is seen in the lungs of adult mice exposed to endotoxemia by intraperitoneal injection of LPS [34]. Next, we assessed the distal lung expression of NF*κ*B target genes previously associated with APAP-induced hepatic injury (*Mmp9*, *Ccl2*, *Il1b*, *Nfkbia*, *Gclc*, and *Ptgs2*). Consistent with pulmonary NF*κ*B activation, by 8 hours of APAP exposure, the expression of all genes was significantly increased compared to baseline (Figure 6(c)).

### 3.6. Toxic APAP Exposure Induces Endoplasmic Reticulum Stress in the Distal Lung

**Figure 7:**
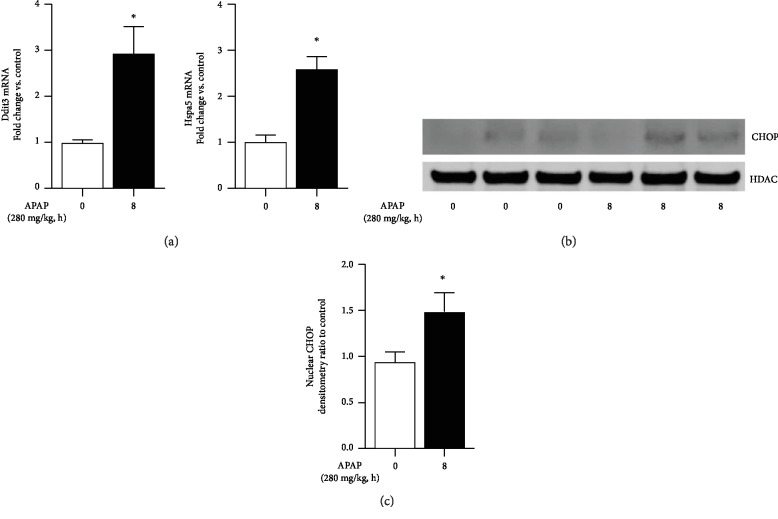
Toxic APAP exposure induces endoplasmic reticulum stress in the distal lung. (a) Fold change in mRNA expression of endoplasmic reticulum (ER) stress markers Ddit3 and Hsps5 in the distal lung after APAP exposure (8 hours; 280 mg/kg, IP). Data expressed as mean ± SEM (*N* = 4‐6 per time point). ^∗^*p* < 0.05 vs. unexposed control. (b) Representative Western blot and (c) densitometric analysis of CHOP in distal lung nuclear extracts after APAP exposure (2, 4, and 8 hours; 280 mg/kg, IP). Data expressed as mean ± SEM (*N* = 4‐6 per time point). ^∗^*p* < 0.05 vs. unexposed control.

Following APAP administration, the liver demonstrates evidence of endoplasmic reticulum (ER) stress [35, 36]. Interrupting this process attenuates APAP-induced liver injury [37]. While we could find reports that other tissues demonstrate evidence of ER stress following APAP exposure, we could find no such report for the lung [38]. Having demonstrated that the distal lung expresses CYP2E1 and accumulates APAP adducts, we specifically interrogated this tissue for evidence of endoplasmic reticulum stress following toxic APAP exposure. Following APAP exposure, distal lung expression of the ER-stress-related markers *Ddit3* (CHOP) and *Hspa5* (BiP0 increased (Figure 7(a))). Furthermore, we found significant nuclear accumulation of CHOP in the distal lung following exposure to APAP (Figures 7(b) and 7(c)). These data demonstrate initiation of ER stress signaling in the distal murine lung following toxic exposure to APAP. This conformation suggests a means to pharmaceutically treat APAP-induced lung injury.

## 4. Discussion

We found that the lung is susceptible to APAP-induced injury. Similar to previous reports, we found that toxic APAP exposure induced significant injury in the large, conducting airways. Here, we report that the peripheral lung is also subject to the injurious effects of APAP. Histologically, there is evidence of macrophage infiltration and alveolar injury within 24 hours of exposure. These histologic findings correspond with an increase in inflammatory cells in BALF obtained from APAP-exposed animals and a sustained depletion in the reduced glutathione in the lung. Furthermore, we demonstrate that the peripheral lung expresses CYP2E1, expression of CYP2E1 increases after APAP exposure, and that expression can be detected in alveolar macrophages and the pulmonary epithelium. We were able to detect the presence of APAP-protein adducts in the peripheral lung, as well as the activation of ER stress and proinflammatory signaling. Together, these results support the hypothesis that the lung is able to directly metabolize APAP, and that following a toxic exposure, there are measurable implications on oxidant, ER stress, and inflammatory stress signaling pathways. Activation of these responses are temporally related to proximal and distal lung injury. Further work is necessary to determine whether this injury can be prevented through pharmacologic interventions aimed precisely at the lung. However, although suggestive, whether these mechanisms underlie the clinical relationship between APAP exposure and pulmonary dysfunction are unknown.

A large body of literature demonstrates that the lung is particularly sensitive to both acute toxic and chronic APAP exposure. In humans, acute overdose has been associated with ARDS [8], pneumonitis [39], and alveolar injury [40]. Both prenatal and early life exposures have been linked to the development of asthma [3, 5–7], while chronic exposure later in life has been associated with an increased risk of chronic obstructive pulmonary disease [4, 41]. Preclinical data across multiple species robustly support the association between APAP exposure and pulmonary injury. Toxic APAP exposure induces bronchiolar injury in rats and mice and induces an ARDS-like picture in pigs [10–14, 42]. Importantly, pulmonary injury appears to occur independently from (but concurrently with) hepatic injury. Firstly, while APAP-induced hepatic injury is dependent on the fasted status of the exposed animal, APAP-induced pulmonary injury is not [14]. Furthermore, despite a significant reduction in APAP-induced liver injury in mice with a liver-specific knockout of NADPH-cytochrome P450 reductase, pulmonary injury remains consistent [13]. This observation is similar to those reported when APAP-exposed mice were pretreated with the mixed-function oxidase inhibitor piperonyl butoxide [15]. Although pretreatment largely abrogated APAP-induced hepatic injury, pulmonary injury remained consistent and significant [15].

There is biologic plausibility to the premise that the lung directly metabolizes APAP and that pulmonary injury occurs secondary to the accumulation of the toxic metabolites of APAP. The respiratory tract endures near continuous exposure to toxic xenobiotic compounds. As such, the expression of xenobiotic metabolizing enzymes, including CYP2E1 is believed to be quite important [19, 43, 44]. In humans, pulmonary CYP2E1 expression has been reported in the peripheral lung, including the alveolar epithelium and macrophages [25–27]. Interestingly, following APAP exposure, APAP-protein adducts can be detected in the areas of the lung that express CYP2E1 [11, 15, 18, 23, 24]. Of note, pulmonary CYP2E1 expression has been linked to pulmonary dysfunction induced by chronic alcohol ingestion [45]. Whether inhibiting pulmonary CYP2E1 expression would attenuate APAP-induced lung injury remains to be tested.

Recent reviews have confirmed that the mechanisms underlying APAP-induced lung injury are not well understood and would benefit from further study [4]. Furthermore, while previous studies have clearly demonstrated the relationship between APAP-exposure and epithelium injury in the larger airways, very little information is known about the effect of APAP on the cells residing in the distal lung. Multiple potential mechanisms could explain how direct pulmonary metabolism of APAP injures the distal lung. These potential mechanisms include oxidative stress injury and activation of ER stress pathways. Our data support a role played by both oxidative and ER stress in the pathogenesis of APAP-induced lung injury.

Previous studies have shown that pulmonary CYP2E1 activity is a source of oxidative stress [20]. Following APAP exposure, we have shown that CYP2E1 expression increases in the distal lung, a potential source of damaging oxidative stress. Furthermore, we demonstrate that APAP exposure is associated with lung glutathione depletion and increased distal lung expression of the glutamate-cysteine ligase catalytic (GCLC) subunit. Previous studies have demonstrated that whole lung pulmonary glutathione decreases with toxic APAP exposure [12, 46]. Additionally, whole lung GCLC expression increases in response to toxic APAP exposure [47]. However, these data were interpreted with a focus on bronchiolar injury. Of note, GCLC overexpression attenuates APAP-induced liver injury [48], but whether this is true in the lung is unknown. Finally, we add *in vivo* data to compliment previous *in vitro* studies demonstrating that APAP toxicity in isolated type II pulmonary epithelial cells and alveolar macrophages is associated with glutathione depletion [9, 28, 49]. Together, these data support the hypothesis that toxic APAP exposure induces oxidative stress in the distal lung, potentially contributing to injury and pulmonary dysfunction.

Recently, the role of ER stress has been implicated in APAP-induced hepatic injury [36, 38, 50]. However, whether toxic APAP exposure induces ER stress response pathways in the lung is unknown. We provide novel evidence that these pathways are in fact active in the distal lung. In the distal lung, toxic APAP exposure induces the expression of *Ddit3* (CHOP) and *Hspa5* (BIP), as well as CHOP nuclear translocation, all of which are markers of ER stress. While ER stress has been implicated in various lung diseases [51], this is the first report linking toxic APAP exposure to activation of ER stress pathways in the lung. More work needs to be done to understand the contribution of these pathways to the pathogenesis of APAP-induced pulmonary injury.

Our study has several limitations. We did not prove that the pulmonary CYP2E1 directly metabolizes APAP. It is possible that toxic APAP metabolites from the liver travel to the lung causing injury. More direct studies are needed, specifically testing whether pulmonary cell-specific CYP2E1 null models are resistant to APAP-induced lung injury. Previous reports have demonstrated sex-specific differences in APAP-induced hepatic and renal injury [52]. In the current study, we only evaluated pulmonary injury in male mice. Future studies are planned to evaluate whether female mice are equally susceptible to APAP-induced lung injury. Furthermore, we exposed mice to a single toxic dose of APAP, and more work needs to be done to evaluate the effect of repetitive, lower doses. Additionally, while we interrogated inflammatory, oxidant stress, and ER stress pathways, other potential mechanisms may underlie APAP-induced lung injury. Specifically, we did not test whether prostaglandin H synthase metabolism of APAP contributes to pulmonary injury [9, 28]. Finally, we used antibody-based approaches to detect CYP2E1 in the lung after APAP exposure using Western blot, immunostaining, and flow cytometry. Nonspecific binding of our antibody to non-CYP2E1 protein could make interpretation problematic. These limitations must be acknowledged. However, we have provided data to demonstrate that the antibody performs as expected with negative controls and positive controls and shows the appropriate expression pattern across organs and microsomal fractions.

In conclusion, toxic APAP exposure results in significant alveolar injury. This injury is associated with the upregulation of CYP2E1 in the resident macrophage and pulmonary epithelium, as well as the presence of APAP-protein adducts, indicative of direct pulmonary metabolism of APAP. Furthermore, toxic APAP exposure is associated with the activation of oxidant stress, ER stress, and inflammatory response pathways. These findings have clinical implications in our assessment of acute acetaminophen overdose, and require further study in the setting of chronic exposure.

## Figures and Tables

**Table 1:** List of genes and primers used for qPCR analysis.

Target	Assay ID
*Cyp2e1*	Mm00491127_m1
*Il1b*	Mm01336189_m1
*Nfkbia*	Mm00477798_m1
*Gclc*	Mm00802655_m1
*Ptgs2*	Mm00478374_m1
*Ddit3*	Mm01135937_g1
*Hspa5*	Mm00517691_m1
*18S*	Mm03928990_g1
*Mmp9*	Mm00442991_m1
*Ccl2*	Mm00441242_m1

**Table 2:** List of antibodies used for Western blot analysis.

Antibody	Vendor	Catalog number
Anti-CYP2E1	Abcam	ab28146
Anti-GAPDH	Cell Signaling Technology	5174
Anti-APAP	Bio-Rad	0016-0104
Anti-p105	Cell Signaling Technology	4717
Anti-I*κ*B*β*	R&D Systems	AF5225
Anti-I*κ*B*α*	Cell Signaling Technology	4814
Anti-p65	Cell Signaling Technology	8242
Anti-p50	Abcam	ab32360
Anti-HDAC1	Cell Signaling Technology	5356
Anti-CHOP	Cell Signaling Technology	2895

## Data Availability

The data used to support the findings of this study are included within the article.
